# Letter to the Editor in response to Chen et al. 2020

**DOI:** 10.1186/s12931-020-01548-0

**Published:** 2020-11-30

**Authors:** Jaya Ponnampalam, George Seligmann, Tanaya Gandhi, Dilen Parmar

**Affiliations:** University College London Medical School, 74 Huntley S, London, WC1E 6DE UK

**Keywords:** Coronavirus disease 2019, Cytokines, Interleukin-6, Tocilizumab

## Abstract

We would like to comment on the article entitled “Association between cytokine profiles and lung injury in COVID-19 pneumonia” by Li-Da Chen and colleagues, with respect to emerging data regarding the immunopathogenesis of COVID-19. Chen et al. demonstrated the relevance of IL-2R, IL-6 and TNF-α in the cytokine storm and IL-6 as an independent predictor for COVID-19 severity. Del Valle et al. corroborated these findings with regard to IL-6 and disease severity, however, they also showed IL-8 to be of significance. This may be explained by the varying techniques used by the two studies to determine severity. Further studies including critically ill patients and the analysis of mortality rates in this patient cohort would greatly enhance the clinical relevance of these findings. As speculated by Chen et al., early studies on the use of tocilizumab in COVID-19 patients were promising, however, full results from ongoing trials are required to confirm a survival benefit in patients treated with tocilizumab. Moreover, investigating the roles of other pro-inflammatory cytokines and their impact on disease severity could potentially inform novel therapeutic targets.

Dear Editor,

We read with great interest the article entitled: “Association between cytokine profiles and lung injury in COVID-19 pneumonia” by Chen et al. [1]. The authors established that three key cytokines (interleukin (IL)-2R, IL-6, tumour necrosis factor (TNF)-α) were significantly higher in 94 coronavirus disease 2019 (COVID-19) patients with pneumonia in comparison to 12 COVID-19 patients without pneumonia (*p* = 0.002, *p* = 0.016 and *p* = 0.016 respectively). To determine the impact of these cytokines, the authors assessed lung injury through computed tomography (CT) severity score and the ratio of PaO_2_/FiO_2_. The results showed that IL-2R and IL-6 were significantly correlated with these markers of disease severity. However, a statistically significant association was not found between TNF-α and COVID-19 severity.

A study by Del Valle et al. [2] investigated the impact of pro-inflammatory cytokines on COVID-19 severity and survival in 1484 patients in New York City. Disease severity was assessed by generating a severity scale, which included multiple laboratory and clinical metrics such as chest X-ray imaging and oxygen therapy modality. The authors found that IL-6 (*p* < 0.0001) and IL-8 (*p* < 0.0001) levels were correlated with severity. Conversely, Chen et al. [1] found that IL-6 (*p* < 0.001) correlated with COVID-19 severity, as assessed by CT severity score, however IL-8 (*p* = 0.057) did not. These differences could be attributed to the small sample size in Chen et al.’s study (94 patients). Furthermore, Chen et al. [1] only used single parameters to assess disease severity (CT severity score and PaO_2_/FiO_2_ separately), whereas Del Valle et al. [2] combined various metrics to generate a severity score. Further investigations to elucidate the effect that individual cytokines have on markers of COVID-19 severity would be useful for risk stratification and clinical monitoring.

The absence of critically ill patients in the study conducted by Chen et al. [1] means it is difficult to determine whether these cytokines are involved in the pathogenesis of varying COVID-19 severity. Han et al. [3] looked at the presence of inflammatory markers in 102 patients with moderate (n = 42), severe (n = 43) or critical (n = 17) COVID-19 disease; they found that IL-6 levels were significantly higher in critically ill patients compared to those with moderate or severe disease (*p* < 0.001). Whilst the median levels of IL-6 increased by 68% (*p* = 0.004) between moderate and severe COVID-19 disease, there was an increase of 85% (*p* = 0.038) between severe and critically ill patients which supports the trend established by Chen et al. [1]. Additionally, Han et al. [3] demonstrated the relationship between IL-6 and disease severity, and determined that IL-6 was the most sensitive and specific marker for diagnosis of severe and critical disease, however further studies are necessary before this can be employed in practice.

Whilst Chen et al. [1] showed significant associations between particular cytokines and disease severity, it is imperative to assess COVID-19 survival and mortality to gain a better understanding of the disease profile. Del Valle et al. [2] investigated the impact of cytokines on survival. After adjusting for confounders, a competing risk model demonstrated that IL-6 (*p* = 0.0038) and TNF-α (*p* = 0.0152) levels were significantly predictive of survival, and that higher serum levels of IL-6 (HR = 2.47, *p* < 0.0001) and TNF-α (HR = 1.80, *p* = 0.0001) were associated with worse survival. These data indicate the importance that cytokines may have on the disease course and outcome.

Chen et al. [1] highlighted the significance of IL-6 due to its association with COVID-19 severity. In addition, IL-6 was found to be the only cytokine which independently predicted the severity of lung injury in patients with pneumonia (*p* < 0.001). The authors remarked that IL-6 could be targeted with tocilizumab, a humanized monoclonal antibody that blocks soluble and membrane-bound IL-6 receptors, thereby inhibiting pro-inflammatory signalling. Indeed, early studies on tocilizumab have shown promise, with a retrospective cohort study by Guaraldi et al. [4] demonstrating that tocilizumab lowered the risk of requiring invasive mechanical ventilation or death in COVID-19 patients with severe pneumonia (*p* = 0.020). Despite these findings, Hoffmann-La Roche [5] announced that their phase III trial on tocilizumab (COVACTA) had yielded disappointing results at week four. Whilst tocilizumab reduced time spent in hospital (*p* = 0.0370), it did not improve clinical status (*p* = 0.36) nor lower mortality (*p* = 0.9410). However, we await conclusive analyses from COVACTA and the results from the RECOVERY, REMAP-CAP and TOCIVID-19 trials to establish whether tocilizumab is effective in treating COVID-19.

The immune system is regulated through complex interactions between multiple immunological cell types and their soluble products. A significant limitation is that Chen et al. [1] analysed six cytokines (IL-1β, IL-2R, IL-6, IL-8, IL-10, TNF-α). However, other key pro-inflammatory cytokines have been known to play a role in the cytokine storm phenomenon that occurs in COVID-19. De Biasi et al. [6] conducted in-vitro studies to assess the production of pro-inflammatory cytokines by CD4^+^ T cells from eight COVID-19 patients compared to six controls. Of note, IL-17A was produced in significantly higher amounts by COVID-19 positive CD4^+^ T cells vs the controls (*p* = 0.0047). In addition, the authors investigated CD4^+^ T cell polyfunctionality after in-vitro stimulation and successfully demonstrated that COVID-19 patients had more CD4^+^ T cells that were simultaneously producing IL-2 and IL-17A compared to the controls (*p* < 0.01). This highlights the importance that IL-17 may have in the immunopathogenesis of COVID-19. Further studies are needed to clarify the specific functions that IL-17 has in the cytokine storm and consequently on disease severity.

Notwithstanding these limitations, Chen et al. successfully illustrated the relevance of IL-2R, IL-6 and TNF-α in the cytokine storm and IL-6 as an independent predictor for COVID-19 severity. Further studies including critically ill patients and the analysis of mortality rates in this patient cohort would greatly enhance the clinical relevance of these findings. Moreover, investigating the roles of other pro-inflammatory cytokines and their impact on disease severity could potentially inform novel therapeutic targets.

## Data Availability

This letter comments on previously published data.

## Abbreviations

COVID-19: Coronavirus disease 2019
CT: Computed tomography
IL: Interleukin
TNF: Tumour necrosis factor
